# Surgery for ulcerative colitis complicated with colorectal cancer: when ileal pouch–anal anastomosis is the right choice

**DOI:** 10.1007/s13304-022-01250-4

**Published:** 2022-02-25

**Authors:** Francesco Tonelli, Carmela Di Martino, Andrea Amorosi, Enrico Mini, Gabriella Nesi

**Affiliations:** 1 Department of Experimental and Clinical Medicine, University of Florence, Florence, Italy; 2 Department of Health Sciences, University Magna Graecia of Catanzaro, Catanzaro, Italy; 3 Department of Health Sciences, University of Florence, Florence, Italy

**Keywords:** Ulcerative colitis, Colorectal cancer, Mucinous carcinoma, Signet-ring carcinoma, Ileo-anal–pouch anastomosis, Hyperthermic intraperitoneal chemotherapy

## Abstract

Patients with ulcerative colitis (UC) are at risk of developing a colorectal cancer. The aim of this study was to examine our experience in the treatment of ulcerative Colitis Cancer (CC), the role of the ileal pouch–anal anastomosis (IPAA), and the clinical outcome of the operated patients. Data from 417 patients operated on for ulcerative colitis were reviewed. Fifty-two (12%) were found to have carcinoma of the colon (*n *= 43) or the rectum (*n *= 9). The indication to surgery, the histopathological type, the cancer stage, the type of surgery, the oncologic outcome, and the functional result of IPAA were examined. The majority of the patients had a mucinous or signet-ring carcinoma. An advanced stage (III or IV) was present in 28% of the patients. Early (stage I or II) CC was found in all except one patient submitted to surgery for high-grade dysplasia, low-grade dysplasia, or refractory colitis. Thirty-nine (75%) of the 52 patients underwent IPAA, 10 patients were treated with a total abdominal proctocolectomy with terminal ileostomy. IPAA was possible in 6/9 rectal CC. Cumulative survival rate 5 and 10 years after surgery was 61% and 53%, respectively. The survival rate was significantly lower for mucinous or signet-ring carcinomas than for other adenocarcinoma. No significant differences of the functional results and quality of life were observed between IPAA patients aged less than or more than 65 years. Failure of the pouch occurred in 5 of 39 (12.8%) patients for cancer of the pouch (2 pts) or for tumoral recurrence at the pelvic or peritoneal level. Early surgery must be considered every time dysplasia is discovered in patients affected by UC. The advanced tumoral stage and the mucous or signet-ring hystotype influence negatively the response to therapy and the survival after surgery. IPAA can be proposed in the majority of the patients with a functional result similar to that of UC patients not affected by CC. Failures of IPAA for peritoneal recurrence or metachronous cancer of the pouch can be observed when CC is advanced, moucinous, localized in the distal rectum, or is associated with primary sclerosing cholangitis.

## Introduction

Chronic inflammation of the colorectal mucosa, as is typically found in ulcerative colitis (UC), is an ascertained risk factor for the onset of colorectal cancer (CRC). The development of colorectal neoplasia in patients affected by UC is related to several factors: duration of UC, extent of colonic involvement, chronically active colitis from childhood, severity of the first attack, primary sclerosing cholangitis (PSC), and family history of CRC [1–4]. Colitis Cancer (CC) is characterized by younger age of occurrence, higher proportion of mucinous and signet-ring cell histology, more proximal colonic distribution, and higher rate of synchronous lesions in confront to sporadic counterpart.

The progression from inflammation to CRC is a multistep process in which the accumulation of genetic mutations leads to sequential mucosal modifications, gradually moving to low-grade dysplasia (LGD), to high-grade dysplasia (HGD), and finally to cancer. Dysplasia can be frequently a multifocal disease. Often, the dysplastic or early neoplastic lesions arising in the colonic mucosa affected by UC are not easily found at colonoscopy, because they are flat and not dissimilar from the surrounding inflamed mucosa. However, it is also possible that dysplastic lesions are easily detected as a raised mass (DALM: dysplasia-associated lesion and mass), showing either the aspect of pedunculated polyp or velvety plaque, similar, respectively, to sporadic adenomatous or villous polyps. Otherwise, thickening and stricturing lesions can be observed especially in long-standing UC. All these alterations are considered precancerous lesions as they result in an increased risk of cancer. Sometimes, these features of the disease are effectively cancerous stigmata. HGDs harbor cancer in more than 30% of the cases, but also the LGDs can show an infiltrating pattern and a rapid progression in malignancy [5, 6].

Given the uncertainties in the detection and classification of UC-associated neoplastic lesions, the surgical indication and the appropriate timing to colectomy particularly in presence of HGD, LGD, or DALM remain controversial. The personal attitude was to operate any case of colitis associated dysplasia. Restorative proctolectomy (RPC) with ileo-anal–pouch anastomosis (IPAA) is largely adopted also in CC, but if performing IPAA also in UC complicated by an advanced rectal cancer is a controversial point. Based on an experience of more than 30 years, we report today our results in UC patients affected by CC and dysplasia.

## Patients and methods

The clinical records of the patients affected by UC observed at the Digestive Surgical Unit of Careggi University Hospital between March 1987 and December 2011 were reviewed. All UC complicated by carcinoma and submitted to surgery have been included in the study. Age at diagnosis, duration, extent, and activity of the disease, preoperative use of immune-modulators and/or biologics, age at surgery, preoperative diagnosis of dysplasia or cancer, localization of CRC, type of surgery, postoperative complications, and adjuvant chemotherapies have been considered.

The site of the CRC was recorded in right, transverse, left colon, and rectum. Extension of disease was valuated according to Montreal classification [7]. CRC was classified according to TNM-UICC 2009 staging system. Non-adenocarcinoma malignancies discovered in the great colon were also recorded. Patients were stratified by pathological stages groups to examine the effect of surgery and complementary therapies on survival. The survival at 5 and 10 years was evaluated.

Local Ethical Committee approved the protocol of the study. Informed consent was obtained from the patients included in the study.

### Surgical technique

Objectives of surgery were to radically remove all the colon and rectum with adequate lymph-node excision and to maintain fecal continence by performing an ileal pouch–anal anastomosis (IPAA). Contraindications to this procedure were the presence of metastatic cancer or locally advanced cancer. In these evidences, proctocolectomy with definitive ileostomy or palliative procedure was chosen. No preoperative radio or chemoradiotherapy was used in patients affected by rectal cancer. Total mesorectal excision was systematically performed when preoperative assessment documented or raised suspicion of rectal cancer.

Different anastomotic techniques were adopted for reconstructive proctocolectomies of our patients. In our first experience, we performed a handsewn IPAA with mucosectomy of the upper canal. This type of IPAA was subsequently reserved to only cases of distal rectal cancer or mucosal dysplasia in proximity of the anal canal. In the last years of our experience, we routinely adopted a double-stapled IPAA. IPAA using a two-stage procedure was adopted in the majority of the patients. In IPAA patients, topic mesalazine was prescribed daily for controlling the inflammation of the residual rectal mucosa sited between the anastomotic line and pectinate line. Endoscopic surveillance of the IPAA patients was performed every year.

Peritonectomy and HIPEC was performed in young patients with CC associated to diffuse omental and peritoneal carcinomatosis. Mitomycin C was utilized for intraperitoneal chemotherapy.

### Histopathological evaluation

Multiple sections of all the parts of the surgical specimens were made for the evaluation of the inflammatory disease, its extension in the colon, and the presence of areas of dysplasia or cancer. According to the WHO Classification of Tumors of the Digestive System, carcinoma was defined as mucinous adenocarcinoma or signet-ring cell adenocarcinoma when the proportion of this component was superior to 50% of the tumor volume.

### Functional results

The functional results of the patients who underwent IPAA were assessed on the answers to a questionnaire over number of defecations, seepage, use of medications, dietary, social, work, and sexual restrictions, satisfaction of the operation. Quality of life was determined by the CGQL score [8]. The data obtained were compared with those obtained in IPAA patients without CC that had been objected of a previous paper [9] and between CC IPAA with age under and over 65 years.

### Statistical analysis

Data were analyzed using SPSS software (SPSS 17.0 for Windows; SPSS, Chicago, IL, USA). The Kaplan–Meier method was used to evaluate the survival. Mean values with standard deviation and Student’s *t *test were used when appropriate;* p* < 0.05 was accepted as a significant value.

## Results

From March 1987 to December 2011, 417 patients with UC underwent surgery in our institution. Fifty-two of these patients (12%) resulted affected by CC. Population characteristics are listed in Table 1. Before surgery**,** patients have been treated with 5-ASA and steroids, immunosuppressive medications, or biological medications. One of these patients had previously undergone to total colectomy and ileo-rectal anastomosis (IRA) and had developed rectal cancer. There were 19 (36.5%) females and 33 (63.4%) males. Mean age at diagnosis of UC was 30.9 years (range 15–65). Mean age at diagnosis of CC was 53.3 years (range 27–77). The mean disease duration was 16.1 years (range 1.1–30.9).

**Table 1:** Characteristics of 52 patients with ulcerative colitis cancer

Characteristics	Patients *n* (%)
Sex	
Female	19 (36.5)
Male	33 (63.4)
Smoke	11 (21.1)
PSC	1 (1.9)
Mean age at diagnosis of ulcerative colitis (yrs, range)	30.9 (15–65)
Mean age at diagnosis of CRC (yrs, range)	53.3 (27–77)
Duration disease (mean yrs, range)	16.1 (1.1–30.9)
Extension of disease^a^	
E3 (Pancolitis)	35 (67.3)
E2 (left-side colitis)	16 (30.8)
E1 (Proctitis)	1 (1.9)
Disease Activity at surgery	
Low	12 (23.1)
Moderate	27 (51.9)
Severe	13 (25)
Indications to surgery	
CRC	29 (55.8)
HGD	6 (11.5)
LGD	11 (21.1)
Refractory colitis	6 (11.5)

^a^According to Montreal classification

The carcinoma arised in cecum, ascending or transverse colon in 23 (48%) patients, in left colon in 18 patients (38.4%), and in the rectum in another 9 patients (17.3%). In two patients, a double primary CC was found. One or more areas of HGD concomitant to CC were detected in 13 patients; meanwhile, areas of LGD were found in another 12 patients. The histopathological data of the CC are referred in Table 2. The majority of the patients had a mucinous or signet-ring carcinoma. These last types of carcinomas had a significantly different tumoral stage (more stage IV; *p* < 0.03, and less stage I; *p* < 0.02) in confront to the other histological types. Apart from adenocarcinoma, squamous intra-epithelial neoplasia (1 patient) and carcinoid tumors of colon (5 patients) were found at the examination of the surgical specimens. According to TNM-UICC 2009 classification, the stages were the following: Stage I = 18 patients (34%), Stage II = 19 (38%), Stage III = 8 (16%), and Stage IV = 7 (12%) (Table 2).

**Table 2:** Pathological characteristic of the ulcerative colitis cancer

	Total *n *= 52 (%)
Cancer localization	
Rectum	9 (17.3)
Left colon	20 (38.4)
Transverse	11 (21.1)
Right colon	12 (23.1)
TNM-UICC stage	
I	18 (34.6)
II-A	16 (30.8)
II-B	3 (5.8)
II-C	0
III-A	0
III-B	4 (7.7)
III-C	4 (7.7)
IV	7 (13.5)
Histotype	
Mucinous	27 (51.9)
Signet ring	4 (7.6)
Adenocarc	21( 40.3)

Comparison of the indication to surgery with the pathological stage of the CC is shown in Table 3. Early (stage I or II) CRCs were observed in all except one patients submitted to surgery for HGD, LGD, or intractable or refractory colitis. The only patient of these groups that had an unexpected advanced (stage III) cancer has been operated for LGD discovered at a surveillance colonoscopy. Instead, all the patients submitted to surgery with the diagnosis of CC had confirmed the diagnosis and 48% of them had an advanced stage (III or IV) CRC. A significative difference of both the rate of advanced CC (stage III and IV) and early CC (stage I and II) was found between patients submitted to regular endoscopic surveillance as compared to those not submitted to surveillance (*p* = 0.04).

**Table 3:** Pathological stage of Ulcerative Colitis Cancer (TNM-UICC) in relationship to regular endoscopic surveillance and preoperative diagnosis

	I	II	III	IV
Regular endoscopic surveillance				
Yes *n*°32	15 (47%)^a^	13 (40.6%)	1 (3.1%)	4 (12.5%)^a^
No *n*°20	2 (10%)	8 (40%)	7 (35%)	3 (15%)
Preoperative diagnosis				
Refractory colitis *n*°6	2 (33.3%)	4 (66.7%)	0	0
Low-grade dysplasia *n*° 11	8 (72.7%)	2 (18.2%)	1 (9.1%)	0
High-grade dysplasia *n*° 6	2 (33.3%)	4 (66.7%)	0	0
Colorectal cancer *n*°29	5 (17.2%)	10 (34.5%)	7 (24.1%)	7 (24.1%)

^a^Patients underwent regular endoscopic surveillance had an earlier stage of cancer than those who did not (*p* = 0.04)

Thirty-nine (75%) of the 52 patients underwent RPC with IPAA, and 10 patients were treated with a total abdominal proctocolectomy with terminal ileostomy. IPAA was indicated in similar rate in patients aged less or more than 65 years. Seven (18%) patients of 39 IPAA underwent handsewn anastomosis. A stage IV patient with obstructive symptoms underwent palliative surgery consisting in side-to-side ileotransverse anastomosis (Table 4). Pathological features, type of surgery, and follow-up of 9 patients with CC at the rectum are indicated in Table 5. In all but one of these patients, the rectal cancer was locally advanced (T3 or 4) or metastatic. Only 4 patients had a prolonged survival; meanwhile, the other 5 had a pelvic recurrence or a metastatic disease. All these patients were affected by a mucinous or signet-ring cancer. Four patients underwent peritonectomy (two with associated HIPEC) for peritoneal metastasis from mucinous carcinoma (3 patients) or signet-ring carcinoma (1 patient) discovered at time of colectomy or arised 10–15 months after IPAA. The score of peritoneal cancer index changed between 11 and 25. Median overall survival of the patients submitted to peritonectomy was 29 months with 3 years survival of 25%.

**Table 4:** Type of surgery in relationship to the age of the patients and to the stage (TNM-UICC), and site of cancer

	IPAA	End ileostomy	Palliative surgery
Age, *n*			
< 65 37	29 (78.4%)	8 (21.6%)	0
> 65 15	10 (66.7%)	4 (26.7%)	1 (6.2%)
Site of CRC, *n*			
Colon 43	34 (79.1%)	8 (18.6%)	1 (2.3%)
Rectum 9	5 (55.5%)	4 (44.4%)	0
Stage of CRC, *n*			
I 18	18(100%)	0	0
II 19	14 (73.7%)	5(26.3%)	0
III 8	6(75%)	2(25%)	0
IV 7	1(14.3%)	5 (71.4%)	1 (14.3%)

**Table 5:** Characteristics of the ulcerative colitis patients with rectal cancer

Patient	Age at surgery	Duration of UC at time of surgery	Type of surgery	Histopathology	TNM	Stage	Adjuvant chemotherapy	Follow-up
1	50	17	TP + I	MAD	T4N0	II-B	Yes	Dead after 8 yrs, pulmonary mtx
2	55	29	IPAA	AD	T2N0	I	No	Alive at 20 yrs
3	66	23	IPAA	MAD	T3N2	III-C	Yes	After 3 yrs reoperated for pelvic recurrence. Dead after 5 yrs from IPAA
4	43	19	IPAA	MAD	T3N0	II-A	Yes	Alive at 13 yrs
5	57	18	IPAA	MAD	T4N1	III-B	Yes	After 3 yrs pouchectomy for recurrence at IPAA. Dead after 6 yrs from IPAA
6	36	15	IPAA	AD	T3N0	II-A	No	Alive at 17 yrs
7	58	15	IPAA	MAD	T3N2	III-C	Yes	After 10 months pouchectomy for pelvic recurrence. Dead after 14 months from IPAA
8	42	6	TP + I	Signet ring	T4N2M1	IV	Yes	Dead after 2 yrs
9	56	15	TP + I	Signet ring	T3N2M1	IV	Yes	Dead after 10 months

*HGD* High-grade dysplasia, *AD *Adenocarcinoma, *MAD* Mucinous adenocarcinoma, *IPAA* Ileo pouch–anal anastomosis, *TP* + *I* Total proctocolectomy and terminal ileostomy

**Fig. 1:**
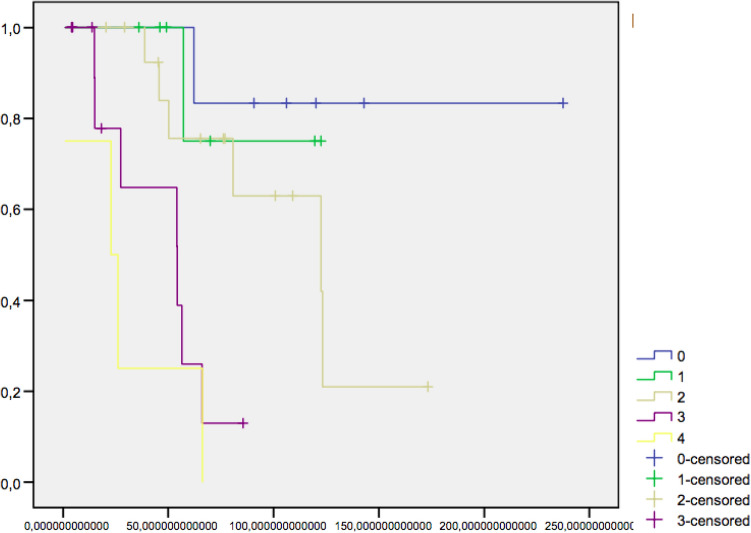
Cumulative Stage I = 0, Stage II-A = 1, Stage II-B = 2, Stage III = 3, and Stage IV = 4

No operative death was observed. Global survival rate of our case series was 122 months as a median, with a 5 and 10 year survival rate of 61% and 53%, respectively. Cumulative survival rate for all the patients and that for the subgroups of patients divided on the base of the pathological stage are shown in Figs. 1 and 2. The cause of death was progression of cancer for a local and/or peritoneal recurrence or for distant metastases. The survival rate at 5 and 10 years from surgery was significantly lower (*p* > 0.01) in patients with mucinous or signet-ring carcinomas than in patients with non-mucinous adenocarcinoma (43% vs 75% at 5 years and 39% vs 70% at 10 years). Two patients developed a metachronous cancer at the level of IPAA. One of them was affected by PSC and showed an advanced cancer inside the pouch 52 months after stapled IPAA. The other patient had a recurrence at the anal transitional zone (ATZ) after a stapled IPAA. The treatment and follow-up of these patients and of other 3 patients who manifested a failure of IPAA due to the local recurrence of the cancer are indicated in Table 6. The removal of the pouch was performed in 5 of 38 (13.1%) patients, more often than in UC patients submitted to IPAA for other indications [7 of 333 IPAA (2.1%) *p* = 0.001]. The IPAA failure (excision of the pouch or maintenance of diversion) was observed globally in 5 of 39 patients (12.8%) and in 3 of the 6 patients (50%) submitted to IPAA for a rectal cancer. The cause of the pouch failure was IPAA cancer (2 patients) or tumoral peritoneal or pelvic recurrence (3 patients). In all but one of these patients, the cancer pattern was mucinous. Mean duration of the pouch in the patients in whom failure occurred was 15 (range 10–52) months.

**Table 6:** Causes of pouch failure in 5/39 IPAA for ulcerative colitis cancer

Patient sex/age (yrs)	Cancer localization	Histotype	Stage	Type of IPAA	Time from IPAA	Causes of failure	Treatment	Follow-up
F/31	Transverse	Mucinous	II (T3N0)	Stapled	52 m	Pouch cancer T4N2M1	Pouch excision	Death 8 months
M/43	Cecum	Mucinous	II (T3N0)	Stapled	17 m	Peritoneal Carcinosis	Ileostomy	Death 2 months
M/66	Distal rectum	Mucinous	III (T3N2)	Handsewn	26 m	Pelvic recurrence	Pelvectomy	27 m
F/57	Proximal rectum	Adenocarcinoma with colloid components	III (T4N1)	Stapled	38 m	Cancer at anastomotic line T3N0	Pouch excision	Death 3 yrs
M/35	Distal rectum	Mucinous	III (T3N2)	Stapled	10 m	Pelvic-peritoneal recurrence	Pouch excision	4 m

An unusual tumoral manifestation was observed in a 31 year old male who was submitted to a stapled IPAA for a mucinous cancer (T2N1) of transverse colon associated with multiple area of HGD in the colon. Area of LGD was present also in the rectum. The pathological examen of the surgical specimen showed a squamous intra-epithelial lesion with a diameter of 0.25 cm surrounded by anal transitional mucosa at the distal margin of the surgical resection. In the following months, biopsies of the mucosa of the anal canal showed dysplastic growth of squamous epithelial in the ATZ. Therefore, 10 months after the first operation, a mucosectomy of the ATZ and a redo-IPAA was performed. The patient maintained a normal function of the pouch in the following years without recurrence of dysplasia or cancer. Considering only the IPAA patients without recurrence of cancer, the functional result and the quality of life evaluated at least 1 year after surgery were not different from those of IPAA operated without CC. No significant differences of the functional results and quality of life were observed between IPAA patients aged less than or more than 65 years. Patients under 65 had diurnal bowel movements of 4.5 (mean, SD 2.4) and nocturnal bowel movements of 0.9 (mean, SD 0.4); meanwhile, patients over 65 had diurnal bowel movements of 4.2 (mean, SD 1.5) and nocturnal bowel movements of 1.3 (mean, SD 0.3). No difference was recorded between these two groups for daily or nightly seepage. The ability to distinguish flatus from stool was present in the 95% of under 65 and in 90% of over 65 IPAA patients. All patients were satisfied with surgery and would recommend it. Work, social, sexual, and dietary restrictions were similar between the two groups. The mean CGQL score was 0.75 (SD 0.11) in under 65 and 0.73(SD 0.14) in over 65 (*p *= n.s.). The patients with handsewn anastomosis had a less-satisfying functional result with imperfect continence in 28% of them, due to nocturnal seepage and/or urgency to defecate.

## Discussion

We observed CC in our UC surgical series in 52 of the 417 operated patients, equivalent to 12.5%. The incidence of CC in the published UC case series is extremely variable from 1.4% to 34% [4]. This so large difference depends by several variables. The factors that weigh most in the event of CC are the duration of the UC and the extent of mucosal inflammation. Analyzing 6 studies that have considered only the pancolitis, the cumulative CRC risk is 2.1%, 8.5%, and 17.8%, respectively, at 10, 20, and 30 years from the diagnosis [4]. The higher incidence of CC is found in gastroenterological, surgical, or tertiary referral centers that make an important selection of the patients. This is also the reason of the rather high incidence of CC in our case series. In the last time, surveillance protocol, endoscopic treatment of dysplasia, timely colectomy, and medical treatment inducing remission of the inflammatory activity have been used more than in the past and had determined less CC [10–12]. A more precise and real estimate of the CC incidence comes from the data of the national cancer registries. For example, the cumulative incidence of CC in the population-based Swedish Registry at 10, 20, and 30 years after diagnosis of IBD is 1%, 1.5%, and 2.7%, respectively [13]. Furthermore, the most recent studies consider for an appropriate evaluation of the CC risk the standardized incidence ratio (SIR) which compares the observed number of CCs to the expected number of CRCs in general population [14]. However, the risk of CC remains very high in UC patients with concurrent PSC. CC develop in 14% and 31% of these patients 10 years and 20 years from the beginning of the disease, respectively [15].

The mean age at diagnosis of CC in our experience was 53.3 yrs similar to that observed in other experiences and significantly earlier than mean age at diagnosis of sporadic CRC [16–19]. Like in other experiences, we had showed an increased proportion of CCs in males in confront of the females. This phenomenon reflects the same protective effect of estrogens invoked for explain the lower incidence of adenomatous polyps and CRC in premenopausal women or in women who had used hormone replacement therapy. The effects of estrogens are mediated by the cellular presence of estrogen receptors (ER) [20, 21].

More the 40% of the CC in our series was located in the right colon. Also, other authors report a similar distribution of CC in UC patients [15, 22–26], but several other authors found more often the carcinoma in the left colon or in the rectum than in right colon [14, 16, 24–26]. This difference could be related to the extension of the colitis: all our CCs found proximally of the splenic flexure were arised in UC pancolitis. An early stage CC was predominant in our case series (> 70%). These data are different from that observed in the sporadic CRC and in some CC series in which almost 50% of CCs were stage I and II and other 50% stage III and IV [17, 19, 27–29], but similar to other authors. They have noticed less-severe tumor stages of the discovered CC in the last 2 decades in confront to the past [2, 11, 16, 26, 30]. These data could depend from increased awareness of the neoplastic risk and a more extensive, and accurate surveillance programs with a good compliance of the patients in the last years in confront of the past. In our experience, the survival of CC patients is 61% and 53% at 5 and 10 years of follow-up, respectively. It is controversial if CC survival is equal, worst, or better in confront of sporadic CRC. The survival of CC is first of all related to the stage at which CC is diagnosed and treated. In many case series, the survival is similar to that of sporadic CRC [22, 24, 26, 31, 32, 34, 35], but in other experiences, it is worse [17, 27, 33, 36] even if a lower rate of advanced stages of CC was observed due to the program of surveillance protocol [37]. In particular, it has been observed that stages III CC had a worse prognosis in confront of sporadic CRC [14, 33], and metastatic CC patients have shorter survival and less benefit from standard chemotherapy than metastatic sporadic CRC patients [38]. The reason of this bad prognosis could be related to the pathological features of CC, different from that of the sporadic CRC, and to the high rate of mucinous and signet-ring cell adenocarcinoma present in CC. Mucinous and signet-ring cell histotypes are rare in sporadic CRC with an incidence, respectively, of 10–20% and 0.9–4%, but are found frequently in our as in other case series and could be responsible of a higher rate of lymph-nodal and peritoneal diffusion and a poorer responsiveness to chemotherapic or radiotherapic regimens [24, 39]. The molecular carcinogenetic process of UC deeply different from that of adenoma–carcinoma sequence usually observed in sporadic CRC can explain this phenomenon. Early events of CC are represented by DNA methylation that induces inhibition of oncosuppressor genes and *MMR* gene (MMR) promoter regions, mutation of *p53*, aneuploidy, and microsatellite instability (MSI). MSI-high (H) was found approximately in more than 30% of UC-associated carcinomas or HGDs [40]. MSI-H/deficient MMR is considered a favorable prognostic factor for nonmetastatic colon adenocarcinoma, but did not show survival advantage in the advanced CRC and is not accompanied to better survival in patients treated with 5-fluorouracil-based adjuvant chemotherapy [41, 42].

To improve patient prognosis, we performed cytoreductive peritonectomies hoping to have a better response to chemotherapy. There is a limited experience of peritonectomy and in particular of HIPEC in the setting of CC. Our experience was rather disappointing, but can be proposed for preventing intestinal obstruction and in young patients.

The discovery of dysplasia at biopsies during a colonoscopy for the surveillance program asks the question if immediate surgery is justified. Meanwhile, the diagnosis of HGD makes easy the decision of operation, because the presence of CC is very high, varying from 42 to 67% [43, 44], and the management of UC with LGD remains a difficult decision. Some centers in which the progression of LGD to advanced lesions (HGD or CRC) or the association with an unrecognized CRC was found extremely high [45, 46], even up to 50% and 20%, respectively, have advocated prompt surgery. Other centers have observed few or no progression of LGD to advanced neoplasia following the patients for many months [47, 48]. A recent metanalysis has been focused on the risk factors for the progression of LGD to CRC. They are LGD in the distal colon, long-standing UC, flat or invisible lesion, diagnosis of dysplasia from an expert gastrointestinal pathologist [48]. Instead, a raised morphology of LGD does not represent a risk factor as these lesions can be more easily discovered, totally resected, and simply controlled at follow-up [49]. Since the estimation of the progression of LGD to an advanced neoplasia is variable in studies published so far, the management of LGD in UC patients remains controversial to date. A randomized prospective multicenter study comparing continuing surveillance vs colectomy at LGD diagnosis with CRC-related death, quality of life, and death from all causes, as primary outcome measures, would be auspicable to resolve this debate, but has not been performed either because a similar study could be criticized for ethical reasons but also for practical difficulties due to validate the best method of identification and characterization of LGD [50, 51]. Until prospective studies on LGD and UC patients will better clarify the real progression of LGD, the indication to surgery should be remain a valid option and its cost–benefit should be showed and discussed with the patients. This direction is confirmed by a recent consensus of ECCO [52].

Given the multiplicity and the possible recurrence of neoplastic lesions, it is inappropriate to adopt partial colonic resections for CC patients, as in the past has been proposed particularly for elderly patients [53, 54]. Even subtotal colectomy with IRA is criticizable, since the presence of colonic carcinoma or dysplasia at time of surgery is an important risk factor for the evidence of cancer in the rectal stump [55]. Therefore, proctocolectomy should be the right choice for CC, but it remains to clarify if is better to do a definitive end ileostomy or to perform RPC. In the last decades, the widely accepted surgical procedure for UC patients requiring surgery has been RPC with IPAA. This procedure was largely adopted also in UC complicated by dysplasia or cancer. The result of IPAA is satisfactory with a good function not different from that achieved for IPAA performed in the absence of CC [32, 56]. We can confirm the good function of the pouch even when the operation was performed in patients more than 65 years old. After all it has been demonstrated that IPAA does not adversely affect quality of life in old patients although daytime and nighttime incontinence is more common than in young patients [57]. However, the failure rate of the pouch performed in UC patients affected by CC can be high varying between 14.2% and 19% [39, 56, 58, 59] and most of the time significantly higher than that observed in IPAA with the absence of CRC. Our experience of pouch failure (12.8%) confirms what other authors have observed. The only exception is the case series of Al-Sukni et al. [32] that have found only a 6% of failure not dissimilar from the failure observed in their experience for IPAA without CC. This good result could be due to the low number of advanced rectal cancer observed and treated with IPAA in their experience. Moreover, how often IPAA is performed when CC is sited in the rectum is not clear, because these data are not indicated in the majority of the published experiences. For example, the most common operation performed at the Mayo Clinic was total proctocolectomy with end ileostomy which was utilized in 51% of the 41 UC patients with rectal adenocarcinoma; meanwhile, IPAA was performed in only 27% of them [60]. In our experience, IPAA was adopted in 66% of the CC sited in the rectum. Seeing the results published in the literature and our personal experience, IPAA performed in the presence of rectal cancer is successful when the tumor stage is favorable with a low rate of failures, whereas IPAA performed for a locally advanced cancer is accompanied by a high rate of failures between 16.5% and 28.5% [58, 60, 61]. The advanced stage of the rectal cancer favors a local recurrence especially if the cancer arises in the rectal middle or lower third. To prevent tumoral recurrence, it would be taken in consideration either pre-operatively or post-operatively a pelvic radiotherapy. Looking to sporadic rectal cancers, preoperative radiotherapy or radiochemotherapy is usually indicated for downstaging T3-4 or N + tumors of the middle and lower rectum. However, this treatment was rarely chosen for rectal cancers arised in UC. Preoperative radiotherapy was chosen in only 11 patients (9 by Wu et al. [39], 1 by Zmora et al. [59], 1 by Gorfine et al. [56]). High rate of severe complications (chronic pouchitis, local sepsis) was observed with the result of pouch failure in 5/11 patients (45%). Even more devastating can be the consequences of the postoperative radiotherapy which inevitably provokes radiation damage to the pouch and the enteric loops dislocated in the pelvis. All seven patients with IPAA who had postoperative radiotherapy had a pouch failure (1 by Remzi et al. [61], 1 by Zmora et al. [59], 3 by Wu et al. [39], 1 by Gorfine et al. [56], 1 by Merchea et al. [60]). We have not adopted radiotherapy neither pre-, nor post-operatively for the fear of exacerbation of the inflammatory disease. The European evidence-based consensus had recommended that radiotherapy should be avoided after IPAA [52]. Conversely, in locally advanced cases, it could be useful to perform radio or radiochemotherapy pre-operatively, and then choosing total proctocolectomy with end ileostomy, since the risk of pouch failure is possible in one of two patients. However, it remains to establish if radiotherapy is able to reduce the local recurrence in advanced rectal tumors arising in UC. Mucinous rectal adenocarcinoma shows a reduced rate of downstaging, an increased rate of positive margin, and poorer overall survival in confront to non-mucinous rectal adenocarcinomas following preoperative chemoradiotherapy [62]. We have frequently found this histopathological type in CCs arising in the rectum and have observed that all the recurrent rectal tumor after total proctocolectomy had a mucinous pattern. The same histotype was considered as a risk factor for the demolition of the pouch in other surgical experience [39].

Another point to be debated is whether performing IPAA for CC could expose to the development of dysplasia or cancer in the pouch. Both the ileal pouch mucosa, that with time undergoes to metaplasia and chronic inflammation, and the residual rectal mucosa below the ileo-anal anastomosis are at risk of developing dysplasia and/or carcinoma. An accurate revision of the IPAA cancers referred in the literature was made by Das et al. in 2007 [63]. They found only 17 *authentic* (i.e., not due to local recurrence of the treated CC or to progression to cancer of the dysplasia that persists at the level of ileo-anal anastomosis) adenocarcinoma in the pouch or in the anorectal mucosa. The majority of these carcinomas are arising from residual rectal mucosa at the level of ATZ. The time intervals from RPCT to the diagnosis of cancer are usually longer than 2 years. The occurrence of pouch cancer following IPAA is very rare. Only two pouch cancers were found by Mark-Christensen et al. [64] over a total of 1723 patients operated for ulcerative colitis at a median follow-up of 12.9 years.

PSC could be another risk factor for pouch cancer onset. UC patients with PSC are at increased risk of CRC either in the intact colon or in the rectal stump after subtotal colectomy and IRA, but the neoplastic risk after RPC has not well defined even if seems a probable event. Imam et al. had followed 65 patients operated on with IPAA for refractory colitis (40%), dysplasia (48%), or carcinoma (10.8%). [65]. The cumulative 5-year incidence of pouch neoplasia was 5.6%. We have observed an advanced cancer of the pouch 4 years after RPC in a PSC-UC patient in whom CC had grown up in transverse colon.

Another point to debate is if the type of pouch anastomosis (i.e., handsewn at the level of pectinate line after mucosectomy of the rectal mucosa of the anal canal, or stapled at the level of the distal rectal mucosa) affects the onset of cancer in ATZ. Several authors have found similar onset of dysplasia or cancer for both anastomotic techniques [32, 66]. No cases of ATZ adenocarcinoma, but only presence of ATZ dysplasia was found in 2.9% and in 3.9% of the 532 stapled IPAA at 10 and 15 years of follow-up, respectively [69]. The discovery of ATZ dysplasia was significantly associated to pathological findings of dysplasia or cancer in the colectomy specimen. Dysplasia of ATZ was managed by mucosectomy or simply controlled depending on the number of positive biopsies and the degree of dysplasia [67]. However, most of the time, ATZ dysplasia and cancer can derive from dysplasia, which was already present in the rectal mucosa when IPAA was performed [63]. Therefore, mucosectomy is recommended when dysplasia or cancer had developed in the distal rectum and intensive endoscopic surveillance with multiple biopsies of ATZ must be taken into account if mucosectomy was not performed in similar cases [68].

Recently, some cases of squamous cell carcinoma (SCC) after IPAA have been showed [69]. The majority of these cancers (five cases) are found in the ATZ zone or in the rectal cuff, but two cases are described in the pouch. These tumors seem to onset by the squamous metaplasia of the rectal or ileal mucosa. The treatment of SCC is referred only in three of the seven published cases and had consisted in chemoradiotherapy. Obviously, this treatment can compromise the function of the pouch, but can be cure the SCC that is extremely sensitive to chemoradiotherapy. We discovered an incidental squamous intra-epithelial neoplasia at the pathological examen of the surgical specimen. This premalignant lesion can progress to invasive carcinoma in HIV positive or in immunocompromised patients. Surgical excision of the anal transitional zone maintaining a functional pouch can avoid the recurrence of the lesion, how it happened in the case we have observed.

## Conclusions

Early surgery must be considered when dysplasia is discovered in patients affected by UC. Long-standing colitis, pancolitis, and sex male favor the development of CC. At least 40% of CC is diagnosed at an advanced tumoral stage and are mucous or signet-ring carcinoma. These factors influence negatively the tumor stage, the response to complementary therapy, and the survival after surgery. IPAA can be proposed in the majority of the patients with a result similar to that of UC patients not affected by CC. Failures of IPAA for peritoneal recurrence or metachronous cancer of the pouch can be observed when CC is advanced, is localized in the distal rectum, or is associated with PSC.
